# Hyperspectral Three-Dimensional Fluorescence Imaging Using Snapshot Optical Tomography

**DOI:** 10.3390/s21113652

**Published:** 2021-05-24

**Authors:** Cory Juntunen, Isabel M. Woller, Yongjin Sung

**Affiliations:** 1 College of Engineering and Applied Science, University of Wisconsin-Milwaukee, 3200 N Cramer St, Milwaukee, WI 53211, USA; juntune3@uwm.edu; 2 College of Health Sciences, University of Wisconsin-Milwaukee, 2025 E Newport Ave, Milwaukee, WI 53211, USA; imwoller@uwm.edu

**Keywords:** hyperspectral imaging, fluorescence microscopy, snapshot tomography

## Abstract

Hyperspectral three-dimensional (3D) imaging can provide both 3D structural and functional information of a specimen. The imaging throughput is typically very low due to the requirement of scanning mechanisms for different depths and wavelengths. Here we demonstrate hyperspectral 3D imaging using Snapshot projection optical tomography (SPOT) and Fourier-transform spectroscopy (FTS). SPOT allows us to instantaneously acquire the projection images corresponding to different viewing angles, while FTS allows us to perform hyperspectral imaging at high spectral resolution. Using fluorescent beads and sunflower pollens, we demonstrate the imaging performance of the developed system.

## 1. Introduction

Hyperspectral fluorescence imaging allows us to interrogate a specimen using multiple fluorophores simultaneously with each fluorophore labeling a different target (e.g., organelles, nucleotides) [1]. It typically uses a broadband light source in tandem with a tunable optical filter to continually scan the entire wavelength range of interest. This contrasts with multispectral fluorescence imaging using 3–5 high-power laser diodes, bandpass filters, or both to acquire the images at a couple of selected wavelengths. The tunable optical filters for hyperspectral fluorescence imaging are compactly modularized, can be attached to an existing microscope, and are more cost-effective than adding multiple laser diodes [2]. When high spectral resolution is required, or the signal is very weak, Fourier-transform spectroscopy (FTS) is typically preferred over tunable optical filters. FTS relies on the Fourier-transform relationship between the spectral profile of light and the interferogram generated with the light for varying optical path differences (OPDs) [3]. With FTS, the spectral resolution below 5 nm can be easily achieved with the value further decreasing as the maximum OPD increases. With FTS, all the wavelength components in the interrogated light contribute to the signal; therefore, the signal-to-noise ratio is higher than with the method using a tunable optical filter.

For a thick specimen, the images corresponding to different depths need to be acquired, as the two-dimensional (2D) imaging provides only a thin slice of the imaged volume. The resultant set of raw images, which contains both the three-dimensional (3D) structural information and one-dimensional (1D) spectral information (or the type of fluorophores), is called the four-dimensional (4D) data cube. Here the 3D structural information refers to the 3D internal structure within a thick specimen, which is distinguished from the 3D surface profile. For the depth-resolved imaging, one can scan the objective focus across the volume of a specimen while recording the images corresponding to different depths sequentially [4]. Alternatively, one can scan a point- or line-focused excitation beam across the volume and map the recorded intensity onto the corresponding location [5]. Whichever method is used, the requirement of a scanning mechanism to acquire the depth information, in addition to the mechanism to scan the wavelength, dramatically increases the data acquisition time for the 4D data cube. Increasing the speed typically requires trading off the spatial resolution, the spectral resolution, or both. In one extreme case, snapshot 4D imaging has been demonstrated at coarse spatial resolution (8.8 μm and 12.6 μm in the transverse and axial directions, respectively) and moderately high spectral resolution (11 nm) [6]. Here, we demonstrate a method of acquiring the 4D data cube at moderately high spatial resolution (about 1 μm) and very high spectral resolution (less than 5 nm). The system is built upon a snapshot optical tomography technique, which captures the 3D volume of a specimen in a single snapshot.

## 2. Related Work

### 2.1. Snapshot Tomography: Snapshot Volumetric Imaging of 3D Internal Structure

For microscopic specimens, various methods have been proposed to image the entire volume of a specimen at the cost of reduced spatial resolution. One approach is to remap different depthts to separate locations on the camera sensor using a volume hologram [7], a distorted grating [8,9], or a liquid-crystal spatial light modulator [10]. Another approach is to record the projection images corresponding to different viewing angles onto the specimen in a single snapshot and reconstruct the 3D internal structure from the recorded projection images. This approach, called light-field microscopy (LFM), is similar to X-ray computed tomography (CT). The original LFM uses a micro-lens array (MLA) to capture the 4D light field (2D intensity and 2D angle) after the specimen, from which the projection images are synthesized through volumetric deconvolution [11]. The second class of LFMs capture the projection images directly by placing the MLA at the pupil plane or the back focal plane of the objective lens [12,13,14,15]. The third class of LFM places the LFM in a 4F telecentric configuration with the objective lens and places an aperture stop at the back focal plane of a relay lens [16]. This configuration is dual telecentric and blocks the high-angle stray rays from outside of the field of view without sacrificing the light collection efficiency. Using the MLA for angular multiplexing of illumination beams instead of deconvolving the light field after the sample has also been demonstrated. This method called Snapshot holographic optical tomography uses defocused imaging and digital holography to separately record the incident beams and restore the sharpness, respectively [17].

### 2.2. Hyperspectral Three-Dimensional Imaging

Numerous studies exist on hyperspectral 3D imaging. Depending on the imaging contrast, different strategies have been used. For example, hyperspectral 3D absorption imaging has been demonstrated by combining the Fourier-transform spectroscopy with sample-rotation tomography using the infrared light [18] and X-rays [19]. In the visible wavelength range, it has been demonstrated using wavelength scan and beam-rotation tomography [20]. Spectroscopic optical coherence tomography has been shown to provide the depth-resolved attenuation coefficients for different wavelengths [21,22]. Hyperspectral 3D refractive index imaging has also been demonstrated using wavelength scan and beam-rotation tomography [20,23]. Hyperspectral 3D fluorescence imaging, which is most relevant to the present work, has been demonstrated using a spectrometer-equipped camera in combination with confocal microscopy [24], light-sheet microscopy [25], and scanning laser optical tomography [26]. Notably, a snapshot method for hyperspectral 3D fluorescence imaging has been demonstrated by trading off the spatial resolution [6], which is distinguished from other snapshot techniques for hyperspectral 3D surface profiling [27,28,29].

## 3. Snapshot Projection Optical Tomography (SPOT) Combined with Fourier-Transform Spectroscopy (FTS) Module

### 3.1. System Design

**Figure 1:**
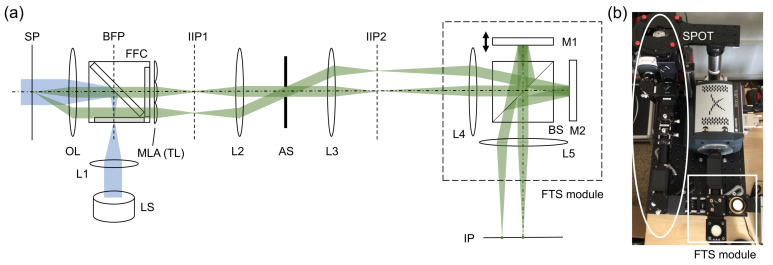
Snapshot projection optical tomography (SPOT) system combined with Fourier-transform spectroscopy (FTS) module: (**a**) schematic diagram, and (**b**) a photograph of the combined system. LS: light source (LED); L1–L5: lenses; M1, M2: mirrors, OL: objective lens; MLA: micro-lens array; TL: tube lens; FFC: fluorescence filter cube; BS: beam splitter; AS: aperture stop; SP: sample plane; IIP1, IIP2: intermediate image planes; and IP: image plane. Two emission beam paths are traced from a point-like emitter at the sample plane to the image plane.

Snapshot projection optical tomography (SPOT) allows us to acquire the projection images corresponding to different viewing angles in a single snapshot, from which a three-dimensional distribution of fluorophores can be calculated using a tomographic algorithm. Figure 1a,b shows a schematic diagram of the SPOT system, which is combined with a Fourier-transform spectroscopy (FTS) module for hyperspectral 3D fluorescence imaging. For the light source (LS), we used a collimated, high-power light-emitting-diode (LED) (Thorlabs, SOLIS-505C) with the peak wavelength of 505 nm and the typical output power of 1.5 W. The excitation light was delivered to the sample plane by the lens L1 (f = 85 mm) and the objective lens (Nikon, Plan Apo VC x60, 1.4 NA). The fluorescence filter cube (Semrock, DA/FI/TX-3X-A-NQF) was inserted between L1 and the objective lens (OL). The emitted fluorescence light was collected using the same objective lens and delivered to the micro-lens array (MLA) using two lenses (not shown) in a 4F telecentric configuration. The relay lenses were introduced to access to the back focal plane of OL and de-magnify the beams by a factor of 2.86. The MLA (Edmund, 64-479), which was used as a tube lens (TL), had the pitch of 500 μm and the focal length of 13.8 mm. Two lenses (L2 and L3) relay the image from the first intermediate image plane IIP1 to the second intermediate image plane IIP2 magnifying the beams by a factor of 4. An iris diaphragm was inserted at the back focal plane of L2, which served as the aperture stop (AS). The overall magnification was 47.3, and the field of view 42 μm. For the wavelength of 530 nm, the diffraction limit was 1.23 μm, when the width of lenslet was used for the calculation. It was 0.87 μm, when the diagonal of the square aperture of lenslet was used for the calculation. The camera pixel resolution was 0.27 μm. To minimize chromatic aberration, we used achromats for all the lenses except for the MLA. The cube beam splitter inserted in the Fourier space (where the beams from a point emitter at the sample plane propagate as parallel beams) did not significantly increase the chromatic aberration, if any. Although the chromatic aberration would still exist, we did not observe the degradation in image quality for the wavelength range used in the experiment. This may be partially attributed to the relatively low spatial resolution of the system.

The FTS module is a Michelson interferometer mounted on a cage system (Figure 1). One of the mirrors (M1) is mounted on a translation stage (Physik Instrumente, P-721.CDQ, Auburn, MA, USA) with the travel range of 100 μm, the resolution of 0.7 nm, and the repeatability of ±5 nm. The stage is controlled in a closed loop using a capacitive sensor and a digital piezo controller (Physik Instrumente, E-709.CR). Two lenses (L4 and L5) were used to deliver the images from IIP2 to the image plane (IP), where a camera was located. The intermediate planes (IIP1 and IIP2) and the image plane (IP) are conjugate to the sample plane (SP). Using the cage system and the precise translation stage, we were able to obtain the FTS spectrum without a translation stage correction, which typically requires installing a separate laser. To record the images, we used an electron-multiplying charge-coupled-device (EMCCD) camera (Andor, iXon Ultra 888, Belfast, UK) with the pixel size of 13 μm.

### 3.2. Data Acquisition

**Figure 2:**
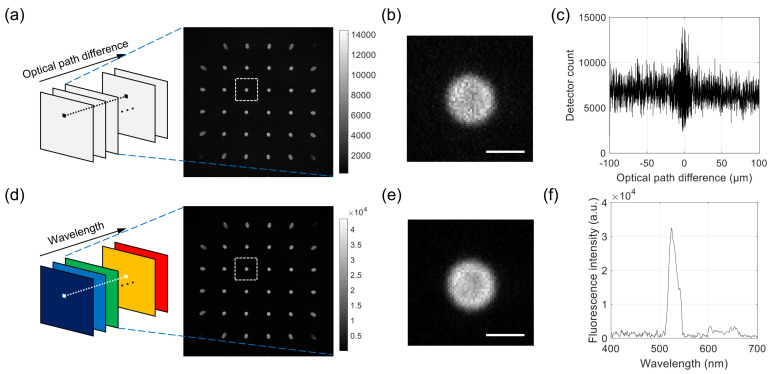
Example raw images acquired with the developed system and Fourier-transform spectroscopy (FTS) data processing. (**a**–**c**) For a 6 μm fluorescent bead, a series of interferogram images is acquired for different optical path differences. Each image consists of a multitude of projection images, as shown in the extracted image on the right. (**b**) shows a magnified image of the rectangular region in (**a**), and (**c**) shows the intensity of interferogram at the center of bead. (**d**–**f**) From the raw images in Figure 2a, a series of hyperspectral images (**a**) is calculated. The extracted image on the right corresponds to the wavelength of 530 nm. (**e**) shows a magnified image of the rectangular region in (**d**), and (**f**) shows the intensity of reconstructed spectrum at the center of the bead. Scale bars in (**b**,**e**): 5 μm.

For each sample, 2000 images were acquired with increasing optical path difference (OPD). An example series of raw images is shown in Figure 2a, with one example image in the stack extracted and shown on the right. The sample was a 6 μm polystyrene bead with the outer layer stained with fluorescent dyes. Each raw image contains a multitude of projection images, one of which is magnified and shown in Figure 2b. The intensity values at the pixel in Figure 2a with the OPD as abscissa are shown in Figure 2c. To record the images, we used the EM gain of 100 and the exposure time of 10 msec. The total data acquisition time was about 140 s. We used LabVIEW (National Instruments, version 15) for synchronous control of the translation stage and the camera.

To check the accuracy of the FTS module, we used two laser sources with different wavelengths: a He-Ne laser (Thorlabs, HNL210LB) and a 488-nm diode laser (Coherent, OBIS 488-60 LS). The FTS module was attached to a custom-built wide-field microscope with the collimated laser beam used for the illumination. Neutral density filters were used to prevent the saturation of pixels. The interferogram for one of the pixels near the center was used to calculate the spectrum of each laser. To check the spatial resolution of SPOT, we acquired a single image for the maximum EM gain of 300 and the exposure time of 0.5 s. A background image was acquired separately and subtracted from the sample image.

The FTS data processing described below was applied to the interferogram at each pixel, which provided the wavelength spectrum at the pixel location. By applying the sample operation to all the pixels, we can obtain a hyperspectral stack of images, each containing a multitude of projection images, as shown in Figure 2d. An example processed image corresponding to the wavelength of 530 nm is extracted from the stack and shown on the right. One of the projection images is shown in Figure 2d, which corresponds to the same projection angle as the one in Figure 2b. Figure 2e shows the spectrum at the pixel location in Figure 2d, which was obtained from the raw interferogram in Figure 2c.

### 3.3. Data Processing

For the FTS data processing, we first used a binary mask to identify each pixel location containing signal from the sample. The intensity values sampled for different OPDs make up the raw interferogram for the pixel, which provides the light spectrum at the location. The interferogram was processed as follows. First, the point where the two beams constructively interfered (zero OPD) was found. Because the interferogram was discretely sampled, the maximum intensity might not occur at the actual zero OPD location. To address this problem, we linearly detrended the data by removing the best straight-fit line, then found the the upper envelope of the interferogram using a curve fitting method, which is implemented as a built-in function in MATLAB (Mathworks, 2020a). Then, the interferogram was shifted horizontally so that the peak of the envelope was located at the center of the OPD axis and shifted vertically so that it had a mean of zero. Next, we applied the apodization. Due to the finite sampling distance, simply applying the Fourier transform to the unweighted raw interferogram returns the true spectrum convolved with a sinc function. As the sidelobes for the sinc function can distort the shape of the reconstructed spectrum, the raw interferogram is typically multiplied with a weighting function before taking the Fourier transform, which is called apodization [3]. Among various choices, the Norton-Beer Medium apodization function resulted in the lowest mean squared error to the ground truth when compared with other apodization functions and without apodization in simulations conducted with our data processing procedure. Computing the Fourier transform via the non-uniform fast Fourier-transform (NUFFT) algorithm resulted in a spectrum at each pixel [30]. The NUFFT was used due to slight nonlinearity in translation stage positioning, resulting in non-uniform sampling locations. In some cases, high frequency noise was observed when accounting for phase correction. For this reason, the phase correction step was bypassed in our procedure.

**Figure 3:**
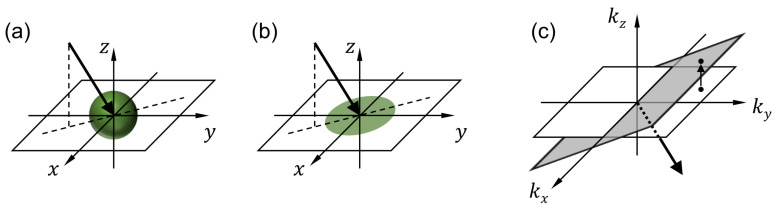
Snapshot projection optical tomography (SPOT) data processing. For each wavelength, the 3D distribution of fluorescence light within the specimen is calculated using the deconvolution and inverse projection operations described in the Methods section. (**a**,**b**) illustrate the projection operation performed by each lenslet in the micro-lens array, and (**c**) shows the inverse projection operation performed in the reconstruction. (x,y,z) are the Cartesian coordinates with z being the optical axis direction. $\left(k_{x},k_{y},k_{z}\right)$ are the spatial frequency components corresponding to (x,y,z), respectively.

For each wavelength, the processed image contains multiple projection images that correspond to different viewing angles. Figure 3a shows the imaging process performed by one of the lenslets. Consider the (m,n)th lenslet, whose center is located at (mp,np), where m,n=−2.5,−1.5,−0.5,0.5,1.5,2.5, and *p* is the lenslet pitch. The image recorded by the lenslet can be written as

(1) $$I^{\left(m,n\right)}\left(x,y\right)=\left(P_{m,n}O\left(x,y,z\right)\right)*h\left(x,y\right),$$

where $P_{m,n}$ and h(x,y) represent the projection and blurring operators, respectively. The projection operator represents the integral of the fluorescence intensity along the arrow direction as shown in Figure 3a. For the (m,n)th lenslet, the arrow direction (i.e., the viewing direction) can be determined with two angles $\alpha_{m}$ and $\beta_{n}$ that the projections of the arrow onto the x−z and y−z planes, respectively, make with the *z* axis.

(2a) $$\begin{array}{c} \alpha_{m}=\tan^{-1}\left\{\frac{mp}{{\left[f_{1}^{2}-\left(m^{2}+n^{2}\right)p^{2}\right]}^{1/2}}\right\}, \end{array}$$

(2b) $$\begin{array}{c} \beta_{n}=\tan^{-1}\left\{\frac{np}{{\left[f_{1}^{2}-\left(m^{2}+n^{2}\right)p^{2}\right]}^{1/2}}\right\}, \end{array}$$

where $f_{1}$ is the focal length of objective lens.

For the 3D reconstruction of the fluorescence intensity, we apply deconvolution and an inverse projection operation. For deconvolution, we apply the Richardson–Lucy method [31,32], which is implemented as a built-in function in MATLAB. For the inverse projection, the Fourier transform of each projection image is projected onto a plane with the surface normal vector parallel to the viewing direction as shown in Figure 2c. The projection images are mapped onto different planes according to the viewing angles. After completing the mapping, the 3D inverse Fourier transform provides the fluorescence intensity distribution. This process of reconstructing the 3D image from a small number of projection images and for a limited angular range is an ill-posed inverse problem [33]. Such ill-posedness can be alleviated using additional constraints about the reconstructed object. For example, the positivity constraint, which enforces the reconstructed fluorescence intensity to be positive as it should be, has been shown to retrieve some information that was missing in the data collection [34,35]. For a more detailed description on the SPOT data processing, the readers are referred to our recent publication [16].

## 4. Results and Discussion

We first checked the accuracy of the FTS module using a He-Ne laser and a diode laser with known center wavelengths (632.8 nm and 488 nm) and sufficiently narrow bandwidths. The reconstructed spectra correctly showed the peaks at 633 nm for the He-Ne laser and 488 nm for the diode laser. The measured full width at half maximum (FWHM) of the laser line was 3.5 nm for the He-Ne laser and 2.0 nm for the diode laser. The spectral resolution of FTS is determined by the maximum OPD and the type of apodization function. For the Norton-Beer medium apodization function, the theoretical FWHMs are 3.4 nm and 2.0 nm at the wavelengths of 632.8 nm and 488 nm, respectively, which match well with the measured values. Then, the spatial resolution of SPOT was measured using a green fluorescent bead with the diameter of 0.5 μm (Thermo Fisher, F13839). From the reconstructed bead tomogram, we selected the horizontal (xy) cross section including the maximum intensity pixel. A one-dimensional intensity profile through the center was fitted with a Gaussian function. The FWHM was measured as 1.0 μm, which may be considered as the transverse resolution of SPOT for the design described in the Methods section. This value is between the diffraction limit calculated with the width of lenslet and that calculated with its diagonal length. The same process is applied to a vertical (xz) cross section, which provided 2.1 μm for the axial resolution.

**Figure 4:**
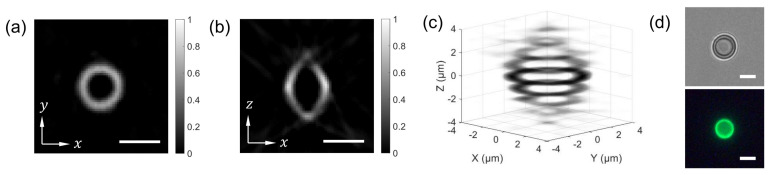
SPOT reconstruction result. For the image shown in Figure 2D, the horizontal (**a**) and vertical (**b**) cross sections of the 6 μm bead are calculated for the wavelength of 530 nm. (**c**) shows a 3D rendered image of the reconstructed bead. (**d**) A bright field (defocused) and a wide-field fluorescence image of the bead. Scale bars: 5 μm.

**Figure 5:**
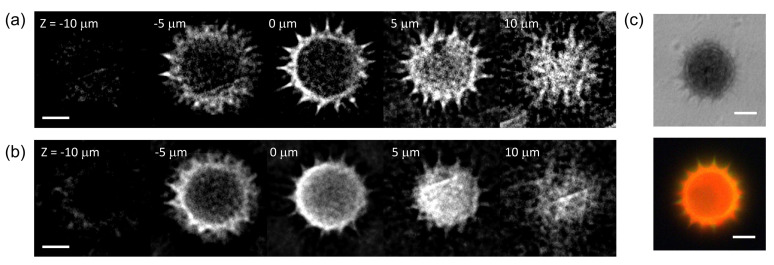
Hyperspectral 3D imaging of a sunflower pollen. (**a**,**b**) show axial stacks of a sunflower pollen reconstructed for the wavelengths of 540 nm and 620 nm, respectively. (**c**) A bright field and a wide-field fluorescence image of the same sample. Scale bars: 10 μm.

The combined FTS-SPOT system was applied to a 6 μm polystyrene bead with the surface layer stained with green, orange, and dark-red fluorescent dyes (Invitrogen, F14806, Waltham, MA, USA). A drop of undiluted bead solution was spread on a microscope slide (1 mm thickness) and covered with a No. 1 glass coverslip. The coverslip was fixed to the microscope slide with tape. For the light source used in this study (peak wavelength 505 nm and bandwidth 42 nm), only the green fluorescent dye was excited. The measured spectrum in Figure 2f shows the fluorescence emission spectrum with the peak at 530 nm and the cut off by the emission filter at 512 nm and 545 nm. Figure 4a,b show a horizontal and a vertical cross section, respectively, of the reconstructed bead for 530 nm. The horizontal cross section shows a ring structure due to the bead surface stained with dyes. This is not visible in the projection images in Figure 2b,e. The width of the ring is blurred due to the finite resolution of SPOT. The vertical cross section clearly shows the optical sectioning capability of SPOT, although the image is elongated along the optical axis (z) direction as with wide-field fluorescence microscopy. Next, we imaged a sunflower pollen grain (Vision Scientific Company, Yuseong-Gu, Daejeon-Si, Korea) using the developed system. The core of the pollen emits green fluorescence light, while the envelope emits red fluorescence light. The two distinctive fluorescence emissions are clearly seen in the axial stacks of the pollen reconstructed at 540 nm and 620 nm, which are shown in Figure 5a,b, respectively. The horizontal cross sections in Figure 5a show the characteristic spiky surface of the pollen, while those in Figure 5b show the round, smooth surface in the core.

FTS relies on the reciprocal relationship between the spectral profile of light input and the interferogram generated using it for various optical path length differences. The translation stage we adopted provides the maximum OPD of 200 µm, which provides the spectral resolution of 60 cm${}^{-1}$ with the boxcar truncation and 84 cm${}^{-1}$ with the Norton-Beer medium apodization function [3]. The maxiumum OPD value, however, can be increased using a different stage, depending on the application. The spatial resolution of SPOT is determined by the numerical aperture of objective lens and the number of projection images, which are recorded together [16]. Increasing the number of projection images reduces the spatial resolution for each projection image, while decreasing it degrades the quality of reconstructed tomogram. There is an optimal number of projection images, which probably depends on the type of imaged specimen.

As SPOT acquires the 3D image in a single snapshot, the imaging speed of the combined method is simply determined by the camera frame rate and the required sampling number to achieve the target spectral resolution for FTS. The maximum frame rate of the EMCCD camera is 26 frames/sec for the full field (1024×1024 pixels). The total data acquisition time to acquire 2000 interferograms is currently about 140 s. Noteworthy, using brighter fluorophores and a strong excitation source, the EMCCD can be replaced with a faster sCMOS camera, which can go over 200 frames/s for the same field of view. With regard to the sampling number for FTS, several approaches have been proposed to reduce the sampling number without sacrificing the spectral resolution. For example, compressed sensing utilizes the fact that most real-world signals can be represented by a set of functions with a fewer number of coefficients. The compressibility prior has been used for various applications including fluorescence microscopy and hyperspectral imaging [36,37]. Another approach is to use deep learning, which utilizes the training data set with known answers [38]. We have demonstrated that a convolutional neural network trained with 30 FTS images can correctly predict three-channel fluorescence images with the sampling number of only 50. Combining deep learning and the method described here is left as our future work.

The developed system can be readily applied to many new applications, as well as any applications currently using fluorescent imaging. In applications currently using fluorescent imaging, the additional spatial information may lead to more accurate classification or even the discovery of a new correlation. In order to fully investigate cellular morphology during cornification, a recent study [39] used eight fluorophores in separate trials, while only capturing 2D images. Since cellular structures naturally form in 3D, it is difficult to fully observe biological phenomena with standard 2D microscopy methods. Using our proposed method, the cornification process could be observed with eight fluorophores simultaneously, and the cell could be reconstructed in 3D, allowing for unprecedented observation information of this process. Furthermore, a recent study [40] which stressed the importance of automated algae classification, used a fully connected neural network to classify six types of algae by measuring the autofluorescent response from six excitation wavelengths. Since the optical system we built captures the 3D spatial information, as well as the spectral information with high resolution, it is likely possible to use our system along with a similar deep learning model to classify several additional types of algae.

## 5. Conclusions

In this paper, we have demonstrated hyperspectral 3D fluorescence imaging by combining Fourier-transform spectroscopy with a snapshot tomography technique. The spectral resolution measured with lasers matches with the theoretical prediction: 2.0 nm and 3.4 nm at the wavelengths of 632.8 nm and 488 nm, respectively. The spatial resolution was measured as 1.0 μm and 2.1 μm in the transverse and axial directions, respectively. Using a fluorescent bead and a sunflower pollen grain, we demonstrated the capability of acquiring a 4D (3D structure and 1D spectrum) data cube. The developed system will be useful for a wide array of applications such as observing biological phenomena with much more available information.

## Data Availability

The data presented in this study are available on request from the corresponding author.

## Abbreviations

The following abbreviations are used in this manuscript:

FTS	Fourier-transform spectroscopy
LFM	Light-field microscopy
SPOT	Snapshot projection optical tomography
MLA	Micro-lens array
OPD	Optical path difference
sCMOS	Scientific complementary metal–oxide–semiconductor
EMCCD	Electron-multiplying charge-coupled-device
NUFFT	non-uniform fast Fourier transform
CT	Computed tomography
